# Thermo-Mechanical Numerical Simulation of Friction Stir Rotation-Assisted Single Point Incremental Forming of Commercially Pure Titanium Sheets

**DOI:** 10.3390/ma17133095

**Published:** 2024-06-24

**Authors:** Marcin Szpunar, Tomasz Trzepieciński, Robert Ostrowski, Krzysztof Żaba, Waldemar Ziaja, Maciej Motyka

**Affiliations:** 1 Doctoral School of the Rzeszów University of Technology, 12 Powstancow Warszawy Ave., 35-959 Rzeszow, Poland; d547@stud.prz.edu.pl; 2 Department of Manufacturing Processes and Production Engineering, Rzeszów University of Technology, 8 Powstancow Warszawy Ave., 35-959 Rzeszow, Poland; 3 Department of Materials Forming and Processing, Rzeszów University of Technology, 8 Powstancow Warszawy Ave., 35-959 Rzeszow, Poland; rostrows@prz.edu.pl; 4 Department of Metal Working and Physical Metallurgy of Non-Ferrous Metals, Faculty of Non-Ferrous Metals, AGH University of Krakow, al. Adama Mickiewicza 30, 30-059 Cracow, Poland; krzyzaba@agh.edu.pl; 5 Department of Materials Science, Faculty of Mechanical Engineering and Aeronautics, Rzeszów University of Technology, 12 Powstancow Warszawy Ave., 35-959 Rzeszow, Poland; wziaja@prz.edu.pl (W.Z.); motyka@prz.edu.pl (M.M.)

**Keywords:** incremental sheet forming, sheet metal forming, single point incremental forming, titanium sheet

## Abstract

Single point incremental forming (SPIF) is becoming more and more widely used in the metal industry due to its high production flexibility and the possibility of obtaining larger material deformations than during conventional sheet metal forming processes. This paper presents the results of the numerical modeling of friction stir rotation-assisted SPIF of commercially pure 0.4 mm-thick titanium sheets. The aim of this research was to build a reliable finite element-based thermo-mechanical model of the warm forming process of titanium sheets. Finite element-based simulations were conducted in Abaqus/Explicit software (version 2019). The formability of sheet metal when forming conical cones with a slope angle of 45° was analyzed. The numerical model assumes complex thermal interactions between the forming tool, the sheet metal and the surroundings. The heat generation capability was used to heat generation caused by frictional sliding. Mesh sensitivity analysis showed that a 1 mm mesh provides the best agreement with the experimental results of total forming force (prediction error 3%). It was observed that the higher the size of finite elements (2 mm and 4 mm), the greater the fluctuation of the total forming force. The maximum temperature recorded in the contact zone using the FLIR T400 infrared camera was 157 °C, while the FE-based model predicted this value with an error of 1.3%. The thinning detected by measuring the drawpiece with the ARGUS non-contact strain measuring system and predicted by the FEM model showed a uniform thickness in the drawpiece wall zone. The FE-based model overestimated the minimum and maximum wall thicknesses by 3.7 and 5.9%, respectively.

## 1. Introduction

Due to their unique properties, titanium sheets are used in the aviation [1], automotive [2], chemical [3] and defense [4] industries. Titanium panels are also used in building engineering [5]. Titanium is distinguished by its high mechanical strength and corrosion resistance, with a low density (4507 kg/m^3^). Constant contact with oxygen causes the formation of a thin (3–7 nm) layer of oxides (mainly TiO_2_) on titanium surfaces, further increasing their resistance to environmental conditions and sea water [6]. Titanium is biologically inert and biotolerant to the human body [7].

Titanium shows very good plastic properties at ambient temperature [8]. The properties of titanium sheets depend mainly on the amount of impurities. An increase in the amount of impurities leads to an increase in hardness and strength properties and a decrease in plastic properties [6]. Technically pure titanium is not heat treated and is only strengthened by the work hardening phenomenon during cold forming. Titanium sheets can be joined to titanium alloys using tailor-welded blanks technology that ensures cost reduction, total weight reduction and an improvement in corrosion behavior thanks to the elimination of overlapping blanks. Such sheets can be processed using sheet metal forming methods [9]. Combined with the continuous development of the latest technologies, titanium sheets are used in various ways in increasingly diverse industries. This requires the development of tools and an adaptation of the forming process parameters to the specific properties of titanium and titanium alloy sheets [10,11]. Conventional deep-drawing processes have been used for many years to form components with complex shapes in cold [12] and hot forming [13] conditions. One of the basic phenomena limiting the formability of materials in deep-drawing is friction [14,15]. Akamatsu et al. [16] reported that lubricants have great effects on the material formability and finished surface. However, pure titanium sheets treated with a heat-induced oxide coating are not in direct contact with the die during forming due to the existence of the oxide layer [17]. The increase in industrialization and the popularization of numerically controlled machine tools at the beginning of the 21st century resulted in the development of the single point incremental forming (SPIF) method proposed in 1967 by Leszak [18]. In SPIF, conventional tools adapted to the shape of the formed component are not required. Forming is carried out by the action of a rotating pin moving gradually along a programmed trajectory adapted to the shape of the component [19]. Many varieties of incremental sheet forming have been developed, such as two point incremental forming, contactless (water jet) incremental forming and electromagnetic incremental forming; however, the concept of forming the material using local small plastic deformations is still valid in these methods [20]. Due to the rotational movement of the tool and the feed movement at the same time, the contact conditions in SPIF are more severe than in conventional sheet metal forming (SMF) processes [21]. SPIF has many advantages related to the flexibility of processing in terms of the shapes of formed components, which is especially important when processing custom-made implants for the individual patient [22]. Nevertheless, the large-scale applications of SPIF are restricted due to their much longer processing time than during conventional SMF [23]. This disadvantage can be compensated by the fact that in SPIF much larger deformations can be obtained without the risk of wall cracking [24] and the forming forces are much lower than during conventional SMF [25]. The technological parameters affecting the possible use of SPIF are tool rotational speed [26], tool diameter [27], lubrication conditions [28] and step size [29]. In SPIF, solid or liquid lubricants that form a protective layer between the sheet and the tool, limiting the metallic contact of the tool and workpiece [30], are usually used.

Previously published works focus on many aspects of the influence of forming process parameters on the surface quality of formed components, geometric accuracy of drawpieces, sheet metal deformability and changes in material properties due to plastic deformation. Most of this work involves experimental research. Shafeek et al. [31] investigated the failure mechanism and formability of commercially pure (CP) Grade 2 titanium sheets in multipoint SPIF (MSPIF). It was found that MSPIF can significantly speed up the time required to produce a component compared to SPIF. Uheida et al. [32] found that the forming force and temperature in SPIF of commercially pure Grade 2 titanium (CP-Ti Gr 2) sheets were directly related to the tool rotation speed. Hussain et al. [33] experimentally studied the formability of CP-Ti Gr 2 sheets in SPIF through different tool rotational speeds that ranged from 450 rpm to 15,000 rpm and feed rates that ranged from 625 mm/min to 10,000 mm/min. They observed a significant increase in temperature (up to approximately 350 °C), with an increase in the feed rate and tool rotational speed. Ambrogio et al. [34] found that increasing the feed rate does not affect the microstructure of the CP-Ti Gr 2 SPIFed component. Nguyen et al. [35] conducted hot SPIF on CP-Ti Gr 2 titanium sheets pre-heated to a temperature of 600 °C. The geometrical accuracy of SPIFed components has been increased compared to experiments at room temperature. Hussain and Al-Ghamdi [36] proposed the application of plasma electrolytic oxidation to deposit a porous coating on the Ti sheet to hold solid lubricant at the tool/sheet interface in the SPIF process of CP titanium sheets. It was found that the developed coating can withstand a range of forming loads and the temperature involved in the SPIF of CP titanium sheets. Behera et al. [37,38] formed the basic geometric shapes using SPIF to characterize the dimensional inaccuracies of CP Grade 1 titanium sheet parts. Analysis of experimental results using the multivariate adaptive regression spline has shown that the developed model was reliable to predict dimensional deviations in SPIFed drawpieces. Sadiq et al. [39] experimentally investigated the effect of tool diameter and step size on the forming angle and fracture depth of incrementally formed CP Grade 1 titanium sheets. The results indicated that decreasing the diameter of the tool and the step size causes an increase in the depth of the fracture. Moreover, an increase in the wall angle caused an increase in the formability of the material.

Most of the previous works are focused on experimental and numerical studies of the formability of titanium alloys sheets. Meanwhile, numerical studies of CP titanium sheets are not widespread. Pande and Barve [40] presented the results of finite element-based numerical studies on the forming of CP Grade 4 titanium sheets. Adamus et al. [41] numerically simulated the SPIF of CP titanium (Grade 1, Grade 2 and Grade 3) thin-walled panels using the finite element method. However, the above-mentioned works did not analyze the influence of friction stir rotation-assisted heating of the workpiece on the formability of titanium sheets. Therefore, this article presents a thermo-mechanical finite element-based model of the SPIF of CP-Ti Gr 2 drawpieces. CP-Ti Gr 2 combines superior corrosion resistance with moderate strength and excellent formability. Due to their peculiar properties, CP-Ti Gr 2 sheets are used in aerospace (brackets, airframe skins), marine (offshore structure, propellers) and chemical (reaction vessels, tube headers) applications. Because it is non-toxic and has a high strength-to-weight ratio, CP is used in the medical field. The numerical research is an extension of the experimental investigations presented in the authors’ previous articles. The values of the components of forming forces, the evolution of the sheet metal temperature as a result of friction stir rotation-assisted tool interactions, the value of plastic strains and the sheet thickness were analyzed.

## 2. Materials and Methods

### 2.1. Material

The test material was 0.4 mm-thick CP-Ti Gr 2 sheets (Timet, Warrensville Heights, OH, USA). CP-Ti Gr 2 at service temperature (up to 882.5 °C) has a hexagonal close-packed crystal structure.

Chemical composition (in wt.%) of CP-Ti Gr 2 sheets provided by Timet is as follows: O—0.23, Fe—0.12, C—0.009, N—0.009 and Ti—balance. The basic mechanical properties of the sheets (Table 1) were determined at room temperature (20 °C) and at elevated temperatures (90 °C and 160 °C). Properties at room temperature were determined using a Z030 testing machine (Zwick/Roell, Ulm, Germany) in accordance with EN ISO 6892-1 [42]. The static uniaxial tensile tests at elevated temperatures were determined using an Instron 8801 (INSTRON, Norwood, MA, USA) testing machine in accordance with the ASTM E21-20 [43] standard. Three samples were tested in each condition and the average value of mechanical parameters was determined.

### 2.2. Experimental Setup

**Figure 1:**
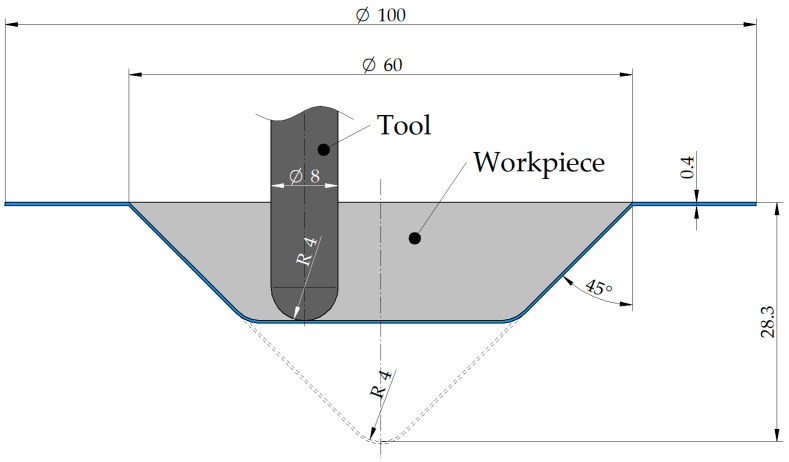
Geometry and dimensions (in mm) of the conical drawpieces.

The formability of sheet metal when forming conical cones was analyzed, which is the basic benchmark test for assessing the formability of the sheets in the SPIF [44,45]. The diameter of the base and height of drawpieces was 60 mm and 28.3 mm (Figure 1), respectively. The slope angle was 45°.

**Figure 2:**
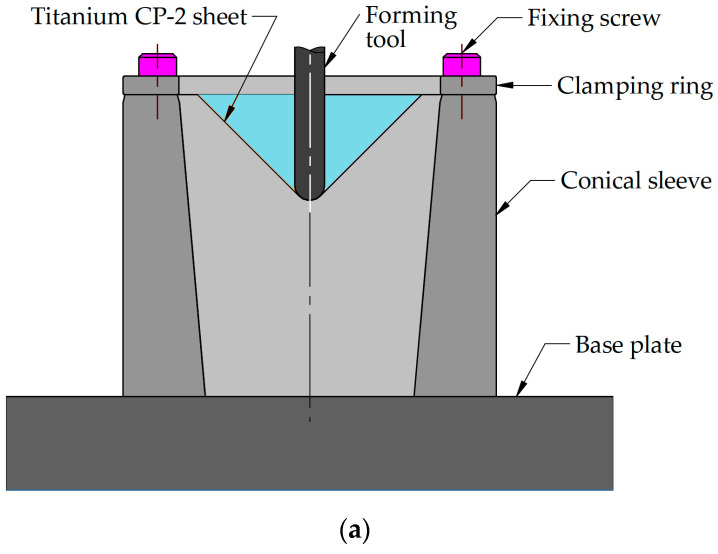

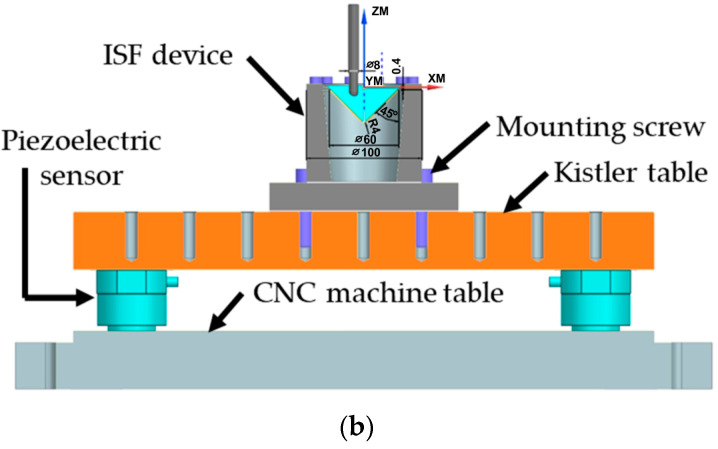
(**a**) schematic diagram of the experimental device and (**b**) test stand.

Workpieces in the form of discs with a diameter of 100 mm were attached between the body and clamping plate using 11 screws evenly distributed around the circumference of the edge of the disc (Figure 2a). The body of the experimental device was mounted in the milling table of the PS95 CNC milling machine (Makino, Tokyo, Japan) through a multi-component force plate of a piezoelectric dynamometer (Figure 2b). The piezoelectric dynamometer (Kistler, Winterthur, Switzerland) type 9366CC allowed for measurement of the forming force components in horizontal (x- and y-axes) and axial (*z*-axis) directions with a sample rate of 200 kHz.

**Figure 3:**
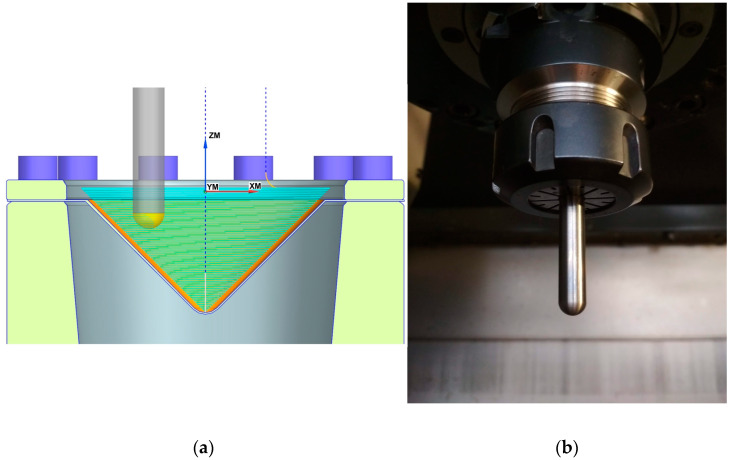
(**a**) tool path trajectory and (**b**) forming tool.

The unidirectional helical trajectory-based tool path strategy (Figure 3a) with a step size of 0.5 mm was considered. A forming tool rotating at 600 rpm gradually indents into the workpiece according to the tool path trajectory generated using the NX CAM program (Siemens, Munich, Germany). The forming tool feed rate was 2000 mm/min. A tungsten carbide grade ISO K30-K40 [46] forming tool in a cylindrical shape with a hemispherical tip (Figure 3b) with a radius of 4 mm was used. Due to the severe contact conditions associated with a small contact area and therefore high contact pressures, it is necessary to lubricate the sheet metal surface. Lubrication reduces resistance to friction [47] and improves the quality of the internal surface of SPIFed components [45]. A 75W-85 gear oil (Castrol Ltd., Liverpool, UK) was used as lubricant [48].

### 2.3. Optical Forming Analysis

**Figure 4:**
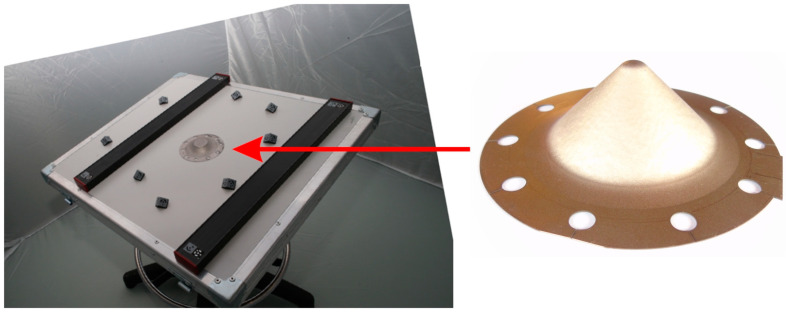
Measuring position for non-contact measurement of the geometry of drawpieces (red arrow indicates the position of the drawpiece on the measuring table).

The wall thickness distribution of the conical drawpiece was determined using a material-independent and non-contact photogrammetric strain measurement ARGUS (GOM Gmbh, Braunschweig, Germany) system. The idea of measuring the local deformations of sheet metal is to measure the coordinates of characteristic points on the tested object. The strain measuring accuracy is up to 0.01% [49]. In order to spatially orient the measured component, coded points are used (Figure 4), which are photographed from many camera shots. The place where the photographs were taken is identified based on control points placed in a given shot.

### 2.4. Temperature Measurement

**Figure 5:**
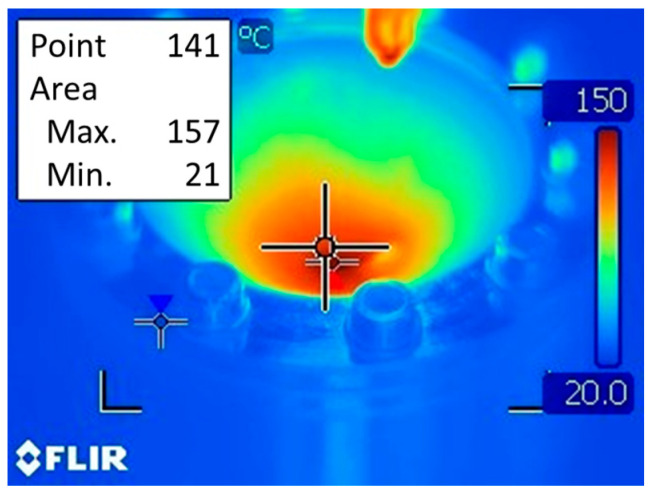
Temperature measurement at the end of forming process.

The FLIR T400 infrared camera (FLIR Systems, Wilsonville, OR, USA) was used to measure the temperature of the sheet metal during the experiments. The measurement at the end of forming was recorded at 157 °C with the thermal imaging camera (Figure 5).

### 2.5. Numerical Modeling

**Figure 6:**
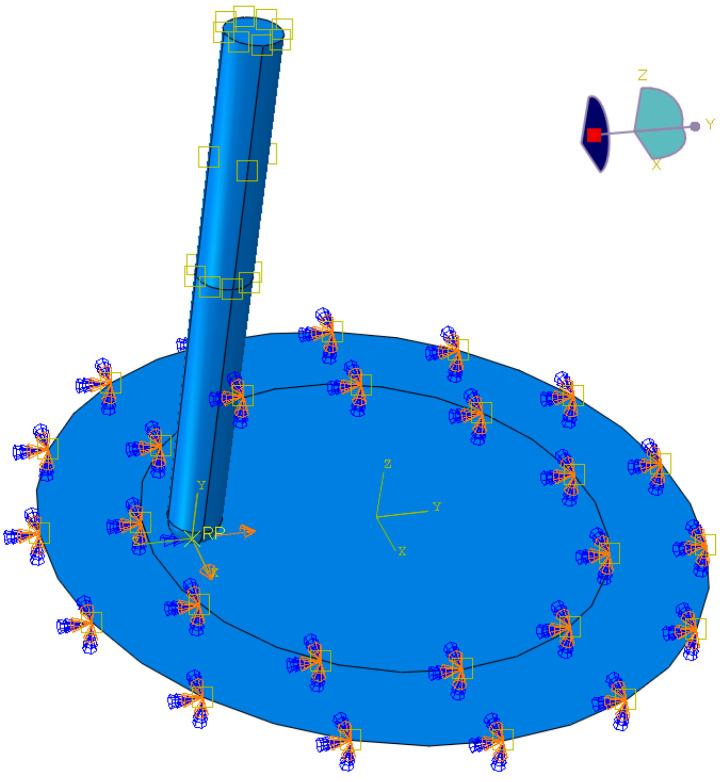
Boundary conditions (yellow squares symbolically mean that a predefined field (temperature) has been applied to the surface, blue cones symbolically mean that model displacements in the X, Y and Z directions are fixed).

Numerical modeling of friction stir rotation-assisted SPIF of CP titanium sheets was performed in the Abaqus/Explicit (Dassault Systemès, Waltham, MA, USA) software (version 2019). An explicit integration scheme is used to calculate the state of a given system at a different time. The surface model of the sheet metal in the form of a disc was fixed on the edge (Figure 6), in the area of the influence of the blankholder in experimental conditions. A tool path exported from Siemens NX CAM version 1938 software (Siemens, Munich, Germany) has been assigned to the forming tool, which was considered as a solid part. The surface-to-surface penalty contact type with the Coulomb friction model was assumed between the surfaces of the sheet metal and forming tool. The heat generation capability was used to heat generation caused by frictional sliding.

**Figure 7:**
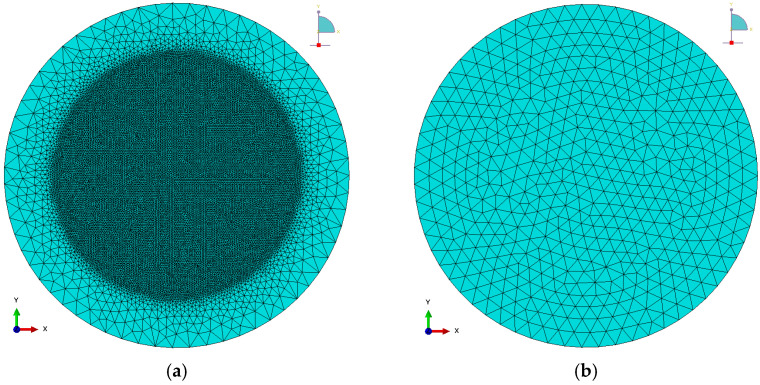
Finite element mesh of sheet metal with dimensions (**a**) 0.5 mm and (**b**) 4 mm; (red and green arrows in the lower left corner indicate the direction of the X and Y axes, respectively; the blue circle indicates the Z axis).

Four-node thermally coupled tetrahedrons were used to discretize the rigid forming tool. Sheet material was meshed using a 3-node thermally coupled triangular shell elements, taking into account the bilinear distribution of temperature and finite membrane strains. During mesh sensitivity analysis, different sizes of finite elements were considered in the deformation zone of the sheet metal: 0.5 mm (Figure 7a), 1 mm, 2 mm and 4 mm (Figure 7b). The numbers of finite elements in various dimensional strategies of finite elements were 36,659, 10,365, 3271 and 1029 for mesh sizes of 0.5 mm, 1 mm, 2 mm and 4 mm, respectively.

**Figure 8:**
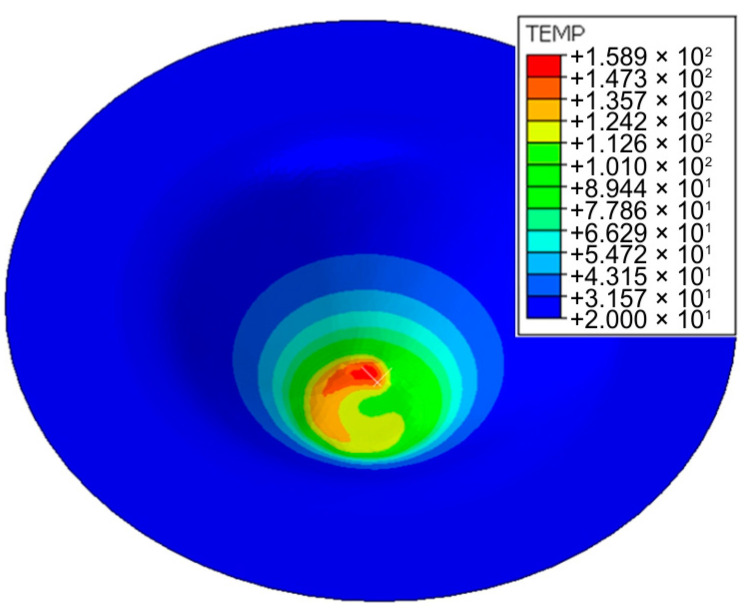
Distribution of temperature at the end of forming process (FEM).

An elastic–plastic model of the sheet metal material was assumed. The temperature in the contact interface generated in friction rotation-assisted SPIF did not exceed 160 °C. The measurement at the end of forming was recorded at 157 °C with the thermal imaging camera (Figure 5), while it was 159 °C in the finite model analysis software (Figure 8).

**Figure 9:**
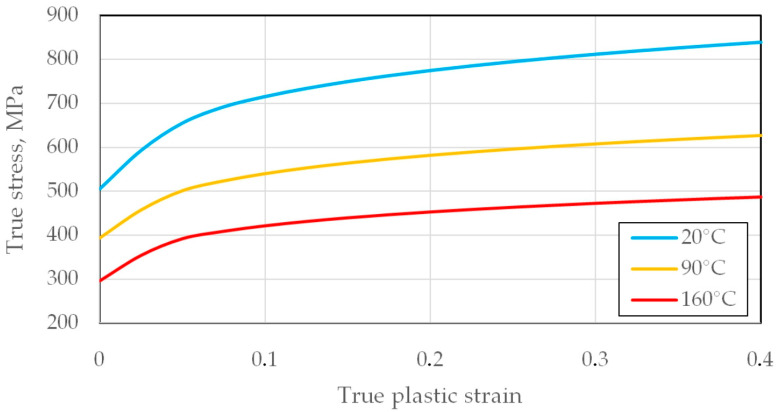
Flow curves of CP-Ti Gr 2 sheets.

Due to the maximum recorded temperature in the contact zone, the simulation included flow curves (Figure 9), prepared in accordance with the approach proposed by Naranjo et al. [50], for temperatures of 20 °C, 90 °C and 160 °C. The remaining physico-mechanical parameters used in the FE-based numerical model of friction stir-assisted SPIF are listed in Table 2. The conditions for heat transfer and convection between the sheet and tool materials and the surroundings were determined by specifying emissivity e = 0.5, convective heat transfer coefficient h = 100 W·m^−2^·K^−1^ and sink temperature T_s_ = 20 °C.

Experimental tests described in the Section 2.2 were carried out to validate the results of numerical studies.

## 3. Results and Discussion

**Figure 10:**
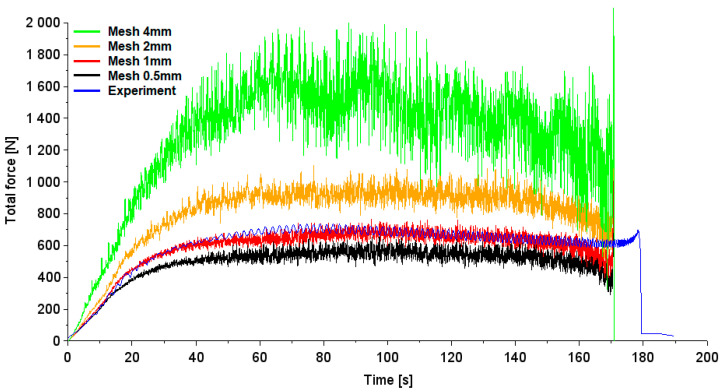
Comparison of the variation in total forming force F for different mesh sizes and experiments.

Figure 10 shows the variation in the total force depending on forming time. Total force F has been determined based on in-plane force F_xy_ and vertical (axial) component F_z_:

(1) $$F=\sqrt{F_{xy}^{2}+F_{z}^{2}}$$

where F_xy_ is the in-plane component of the forming force (F_x_ and F_y_ are horizontal components of force F):

(2) $$F_{xy}=\sqrt{F_{x}^{2}+F_{y}^{2}}$$

The average experimentally determined forming force for the stabilized range is equal to 600 N (Figure 10). As the size of the finite elements increased, the total forming force increased and for the model containing a 4 mm mesh it was approximately twice as high as in the experiment. The increase in the size of the elements makes it difficult to model in a stable way the deformation of the sheet metal using a non-deformable pin with a small rounding radius. It was determined that a model consisting of finite elements of size 1 mm provided the smallest error in predicting the average experimental total force. The difference in the average values of the experimental and numerically determined (mesh size 1 mm) forming force for the forming time range between 60 and 150 s is 2%. The FE-based model containing elements of size 0.5 mm was characterized by an underestimation of the average total force of 15%. The model containing a 2 mm mesh size overestimated the total forming force by approximately 41%.

In the analyses regarding the average value of the total forming force, the range corresponding to the initial rapid increase in forming force was omitted. This unstable range is related to overcoming the resistance to deformation of the sheet due to strain hardening, as explained by Ambrogio et al. [51]. At this stage, the temperature in the contact zone resulting from the friction of the tool was too low to ensure adequate improvement in the formability of the sheet metal. In the final stage of processing, the forces tended to decrease as a result of significant thinning of the sheet metal and limited intensity of strain hardening [52,53], as a result of increasing the temperature in the contact zone and the high material effort.

For many years, calculation time was one of the criteria in mesh sensitivity analysis. However, with the increase in the efficiency of computational programs and the computing power of central processing units (CPUs), computation time has become a secondary parameter, as long as the analysis is performed within a reasonable time. The analysis of the calculation times of the analyzed models (Table 3) showed that the calculation time (65 min) for a model containing elements with a size of 1 mm can be considered acceptable.

**Figure 11:**
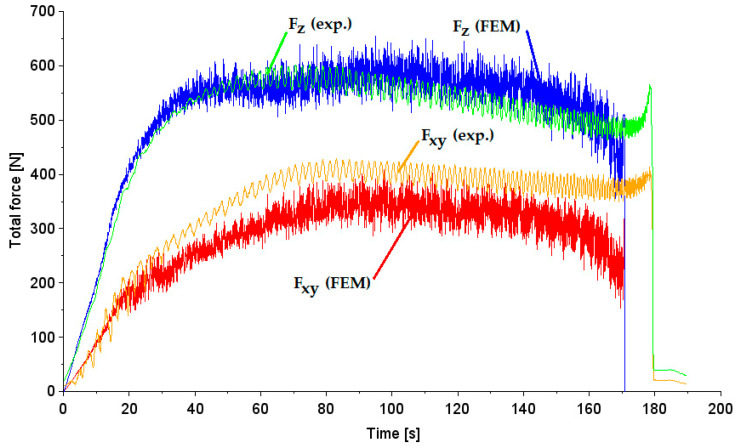
Forming force components (axial and horizontal) in terms of the experiment and FEM (mesh size 1 mm).

The prediction of the value of the axial component of forming force in the SPIF time range of up to 40 s is highly consistent with the experimental course (Figure 11). The average value of axial force in the forming time range between 0 and 160 s is underestimated by the FE-based model by approximately 9%. The in-plane force reaches its maximum for a time of approximately 80 s, i.e., at a drawpiece height of about 8 mm, where the tool has reached contact with the drawpiece. The height of the drawpiece is formed at the expense of reducing the wall thickness. As the height of the conical drawpiece increases, its diameter in horizontal sections decreases, and thus a trend of reducing the in-plane force and axial force is observed (Figure 11). The average value of in-plane force in the forming time range between 0 and 160 s is underestimated by the FE-based model by approximately 14%.

Large fluctuations in the values of the numerically predicted forming force components may result from the changing number of sheet metal nodes that contact the non-deformable tool during the forming process. This conclusion is also reflected in Figure 10. Rosca et al. [54] indicated that the in-plane force oscillates between maximum and minimum values, shifted relative to each other, depending on the position of the forming tool. Formisano et al. [55] concluded that the oscillations are due to a little variability in the stiffness of the sheet, since during a spiral the distance of the tool from the frame is different and, with it, the mechanical reaction of the sheet [55].

**Figure 12:**
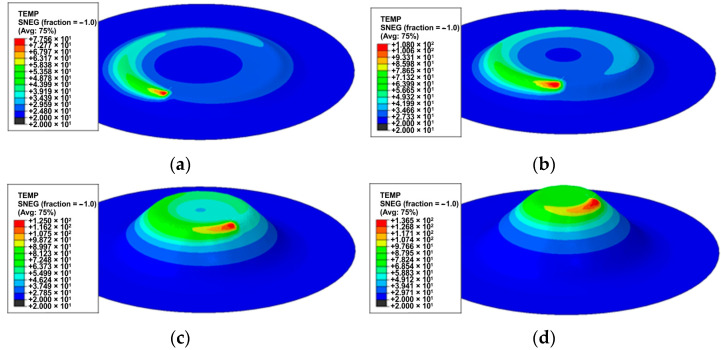
Temperature distribution for drawpieces’ heights: (**a**) 3.7 mm, (**b**) 6.5 mm, (**c**) 14.6 mm and (**d**) 19.5 mm.

Figure 12 shows the evolution in the workpiece’s temperature at selected forming stages. The intensity of material heating in the initial stage of processing is limited due to the long path of tool movement during one revolution along the helical path. During this time, heat is transferred intensively to the surroundings. As the forming time increases, the material temperature in the immediate vicinity of the contact surface increases because the diameter of the tool trajectory decreases at a constant feed rate. At the same time, the temperature distribution becomes more uniform along one circumference of the helical path (Figure 12d).

The value of the maximum temperature of the deformed material at the place of direct contact of the tool with the sheet metal for drawpieces’ heights 3.7 mm, 6.5 mm, 14.6 mm and 19.5 mm is 78, 108, 129 and 136 °C, respectively. It can also be revealed that the heating of the material is local and convection of heat to the surroundings occurs. In the final stage of processing, the temperature of the material of the cone base, deformed at the beginning of the forming process, is close to the ambient temperature (Figure 12d).

Friction stir rotation-assisted SPIF (FSRSPIF) shares similarities with friction stir processing (FSP) and friction stir surfacing (FSS) in terms of localized heat generation through tool friction with the sheet metal [56]. The experimental and FEM analysis reveals that the frictional heat generated by tool rotation at the interface between the tool and the workpiece significantly influences the formability of materials during FSIF. As the tool rotates, frictional heat is produced, softening material in the localized deformation zone and thereby enhancing its formability. In previous findings, a direct correlation between increased tool rotation speeds and improved formability has been found, attributed to the localized frictional heating [48,57]. Furthermore, surface roughness on the tool–sheet contact surface shows variability in both horizontal and vertical directions, which is influenced by the changes in local frictional heating as tool rotation speeds increase [58].

At room temperature, dislocation slip is identified as the primary deformation mechanism for commercially pure titanium (CP-Ti). However, when the temperature decreases to liquid nitrogen temperature (LNT), abundant deformation twins are observed, marking a significant shift in deformation behavior. This study highlights that at temperatures between 20 and 160 °C, dislocation slip continues to dominate as the primary deformation mechanism, confirming the temperature-dependent nature of these mechanisms. The deformation behavior of CP-Ti Gr 2 during FSRSPIF is influenced by temperature variations. At room temperature, dislocation slip predominates, while at LNT dynamic twinning becomes more prominent. This transition from dislocation slip to dynamic twinning with decreasing temperatures is crucial for achieving the outstanding mechanical properties of CP-Ti Gr 2, particularly with the larger grain sizes at LNT. This study underscores that this transition contributes significantly to the material’s mechanical performance during FSRSPIF [59].

**Figure 13:**
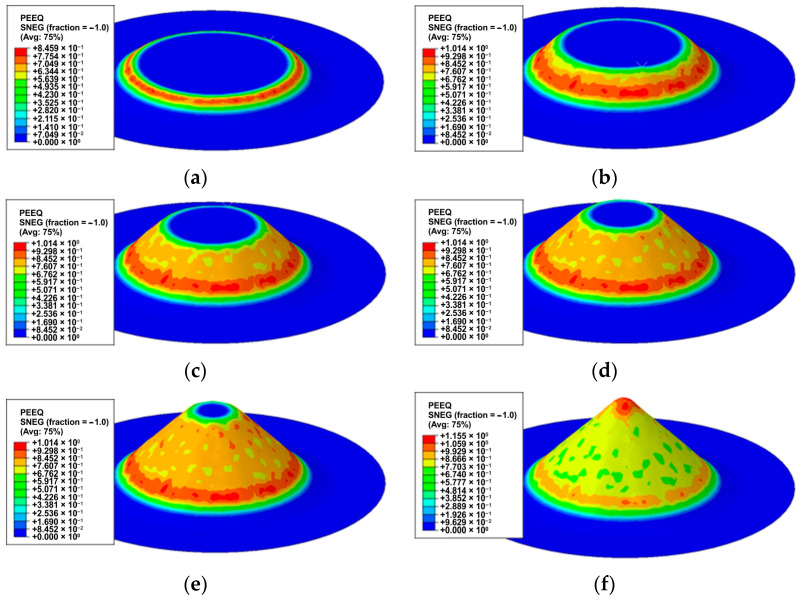
Equivalent plastic strain distribution for drawpieces’ heights: (**a**) 5.8 mm, (**b**) 11 mm, (**c**) 15.2 mm, (**d**) 18.7 mm, (**e**) 23 mm and (**f**) 28.4 mm.

Equivalent plastic strain distribution for selected stages of the forming process is shown in Figure 13. The greatest material strain occurs at the base of the cone, along the first few passes of the tool along the helical path. The drawpiece wall is formed mainly by material deformations occurring directly under the tip of the forming tool. However, the drawpiece wall is formed to a large extent at the expense of thinning the drawpiece wall formed in earlier stages of the SPIF process.

The material in the annular area of the largest equivalent plastic strains (Figure 13a–d) was deformed in the initial stage of forming, when the temperature of the sheet was close to room temperature, with a rapidly increasing total forming force (Figure 10 and Figure 11), necessary to overcome the resistance of the material determined by the yield stress.

**Figure 14:**
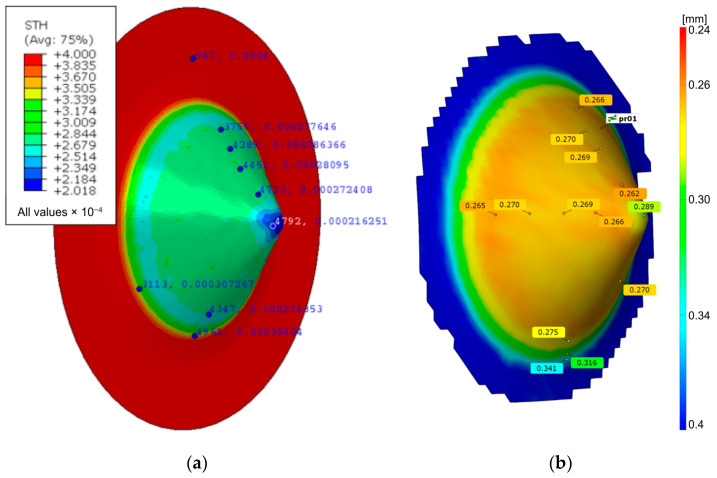
Sheet thickness distribution of a drawpiece achieved using (**a**) the finite element method and (**b**) the ARGUS—a 3D optical strain measurement system.

Figure 14 shows a comparison of the sheet thickness distributions obtained experimentally and by means of non-contact measurement of the strains of the experimental drawpiece. Apart from the base of the drawpiece, the thickness distribution is very even along the forming cone. The nature of the thickness distribution is consistent with the results obtained by Limpadapun and Kesvarakul [59] and Yang et al. [60]. The numerically predicted sidewall thickness is between 0.272 and 0.286 mm (Figure 14a). The sidewall thickness of the experimentally obtained drawpiece varies between 0.262 and 0.27 mm (Figure 14b). In this zone, the prediction of wall thickness is in good agreement with experiment. Locally, the largest difference in the numerical and experimental results occurs locally at the tip of the drawpiece.

## 4. Conclusions

In this article, an attempt was made to create a reliable FE-based model of the pure titanium SPIF process of conical drawpieces, taking into account friction stir rotation-assisted heat generation. The numerical results were experimentally verified by measuring the force parameters and geometry of components determined using the non-contact strain measurement ARGUS system. The following qualitative and quantitative conclusions were found:

(a) The results of mesh sensitivity analysis showed that the predicted total forming force is most similar to the experiment for the numerical model containing a finite element mesh with a size of 1 mm. The average prediction error for the stabilized range of total forming force values was 2%.
(b) It was observed that the higher the finite element size, the greater the fluctuations of the total forming force. This force is determined temporarily based on the contact surface area, i.e., the number of finite element nodes of the sheet metal in contact with the non-deformable tool. Thus, with large finite element sizes, there are large instabilities in the calculations of forming parameters.
(c) As the height of conical drawpieces increases, their diameter in horizontal sections decreases, the material pushing effect decreases, and thus a trend of reducing in-plane and axial forces is observed. At the same time, during friction stir rotation-assisted SPIF, the temperature in the contact zone gradually increases, improving the sheet metal formability.
(d) The maximum temperature observed in the contact zone using the FLIR T400 infrared camera was 157 °C, while the FE-based model predicted this value with an error of 1.3%.
(e) The thinning detected by optical measurement of the drawpiece and predicted by the FEM model showed a uniform thickness in the drawpiece wall zone. The thickness of the side wall of the drawpiece, measured with an optical system with a strain measuring accuracy of 0.01%, varied between 0.262 and 0.27 mm. Meanwhile, the numerically calculated wall thickness was between 0.272 and 0.286 mm. So, the numerical model overestimated the minimum and maximum wall thickness by 3.7 and 5.9%, respectively.
(f) The intensity of material heating in the initial stage of SPIF is limited due to the long path of the tool during one revolution along the helical path and the intense heat dissipation to the environment. As the height of the conical drawpiece increases, the material temperature in the immediate vicinity of the contact interface increases because the radius of the tool path decreases at a constant feed rate. Taking into account that the drawpiece is formed to some extent by the thinning of the previously formed side wall of the drawpiece, it would be beneficial to check how additional hot-air contactless heating or fluid-heating the workpiece will affect the formability of the material. This will be one of the topics of future research.

## Figures and Tables

**Table 1:** Basic mechanical parameters of the CP-Ti Gr 2 sheets.

Test Temperature, °C	Yield Stress, MPa	Ultimate Tensile Stress, MPa	Elongation, %
20	505	651	22.2
90	397	497	42.5
160	298	392	51.4

**Table 2:** Basic physico-mechanical parameters of the tool and sheet metal.

Material	Young’s Modulus, GPa	Poisson’s Ratio	Density, kg·m^−3^	Specific Heat, J·kg^−1^·°C^−1^	Conductivity, W·m^−1^·°C^−1^
Tool	650	0.31	14,450	280	75
Sheet metal	105	0.37	4500	520	19.9

**Table 3:** Mesh setup effect on computation time.

Mesh Size, mm	Number of Elements	Number of Nodes	Computation Time
0.5	36,659	18,362	3 h
1	10,365	5215	1 h 5 min
2	3271	1168	27 min
4	1029	547	15 min

## Data Availability

The data presented in this study are available on request from the corresponding author.
